# Editorial: Rising stars in nutrition and food science technology: Development and utilization of active ingredients in food

**DOI:** 10.3389/fnut.2023.1177158

**Published:** 2023-03-21

**Authors:** Chanchan Sun, Bin Liang, Wenjie Sui

**Affiliations:** ^1^College of Life Sciences, Yantai University, Yantai, Shandong, China; ^2^School of Food Engineering, Ludong University, Yantai, Shandong, China; ^3^State Key Laboratory of Food Nutrition and Safety, Tianjin University of Science and Technology, Tianjin, China

**Keywords:** protein, peptide, polysaccharide, flavonoid, high-tech manufacturing technology

In recent years, people's demand for food is undergoing a transition from quantity to quality and gradually to nutrition. The nutrition of food is not only of great significance to the normal progress of human metabolism, but also plays a key role in the healthy growth, such as defense of viruses and prevention of diseases. The nutritional index of food is the main object of concern in the field of food manufacturing.

In the process of food manufacturing, the nutritional characteristics are mainly controlled by the content of relevant components (nutritional fortifiers, food additives, etc.) and the rational food structure design. Moreover, other challenges associated deal with nutritious foods manufacturing technology and equipment involved. First of all, there is a lack of development in technologies for achieving nutrients with high activity and high absorption rate. Secondly, in terms of equipment, there are still problems related to poor production continuity, large amount of waste water and energy consumption, and poor product quality. The existence of these technical and equipment problems has seriously restricted the healthy development of the food industry and cannot meet people's needs for nutrition.

The papers included in this topic have conducted in-depth research on the regulatory mechanism of active ingredients in food and high-tech manufacturing technology on the nutritional characteristics of food, and have achieved systematic research results. Li Y. et al., Jiang S. et al., Xu et al., and Li X. et al. respectively fermented broccoli by *Lactobacillus strains*, hydrolyzed pig skin collagen with protease, fermented corn milk with probiotics and extracted yeast mannoproteins from *Saccharomyces cerevisiae* to obtain broccoli peptides, pig skin collagen peptides, corn-derived antioxidant peptides and yeast mannoproteins. The study of Li Y. et al. confirmed that broccoli peptides exert anti-inflammatory activity by inhibiting the secretion of inflammatory factors by inflammatory cells. The results of Jiang S. et al. elucidate the interactions between gut bacteria and related cytokines and reveal the mechanisms underlying the anti-Iron deficiency anemia effect of pig skin collagen peptides ferrous chelates. Xu et al. found that IGGIGTVPVGR and LTTVTPGSR isolated and extracted from fermented milk have antioxidant capacity. The findings of Li X. et al. evealed that the prevention of obesity by yeast mannoproteins is highly linked to the promotion of *Parabacteroides distasonis* and inhibition of *Lactobacillus*.

Jiang W. et al., Liu et al., and Zhang et al. respectively established BALB/c mice model, high-fat diet mice model, and anti-oxidation model *in vitro* to confirm the immunomodulatory activity of the polysaccharide obtained from *Craterellus cornucopioides* (Zhang et al.), the advantage in alleviating high-fat diet induced obesity of *Zingiber striolatum* polysaccharides (Jiang W. et al.), and the antioxidant activity of *Houttuynia cordata* polysaccharides (Liu et al.).

Chen et al. found that red-fleshed apple flavonoid extract ameliorated CCl4-induced liver damage by modulating the abundance and composition of intestinal microorganisms in mice. Wang et al. reviewed the nutritional active components of sea buckthorn, such as vitamins, carotenoids, polyphenols, fatty acids, and phytosterols, and their health benefits, such as antioxidant, anticancer, anti-hyperlipidemic, anti-obesity, anti-inflammatory, antimicrobial, antiviral, dermatological, neuroprotective, and hepatoprotective activities. And they revealed the potential of sea buckthorn to be developed into functional foods or dietary supplements for the prevention and treatment of certain chronic diseases (Wang et al.).

Li C. et al. and Zhao et al. respectly investigated effects of drying strategies and steam explosion pretreatment on sporulation and titer of microbial ecological agents with *Bacillus subtilis* and enzymatic digestibility of overground tubers of tiger nut (*Cyperus esculentus* L.). Li C. et al. found that 80°C-hot air drying is an effective drying strategy for promoting sporulation, which improves the titer of microbial ecological agents with *B. subtilis*. The investigation of Zhao et al. revealed that steam explosion pretreatment promoted saccharification of the overground tubers of tiger nut, which paves the way for value-added valorization of the tiger nut plants.

In conclusion, the above research works provide theoretical support for the development and utilization of active ingredients such as protein/polypeptide, polysaccharide and flavonoid in food. This can play a role in improving the quality of both traditional food products and the processing technology, and guiding the investigation of new foods.

## Author contributions

CS drafted the manuscript. BL and WS provided critical review and insight and revised the final version of the editorial. All authors contributed to the article and approved the submitted version.

